# “Healthcare professionals’ attitudes towards euthanasia in the Balearic Islands.”

**DOI:** 10.1186/s12910-025-01262-w

**Published:** 2025-07-19

**Authors:** Daniel Lerma-García, Juan Carlos Muñoz-Camargo, Laura Cervantes-Torres, Sandra Martínez-Rodríguez, María Dolores Onieva-Zafra

**Affiliations:** 1 Department of Nursing, University of Illes Balears, Baleares, Ibiza, Spain; 2 Department of Nursing, Physiotherapy and Occupational Therapy, Ciudad Real Faculty of Nursing, University of Castilla-La-Mancha, Ciudad Real, Spain

**Keywords:** Euthanasia, Attitude, Nurse, Health care professionals

## Abstract

**Background:**

Euthanasia is a topic of ethical, legal, and medical debate, particularly among healthcare professionals, who play a key role in its implementation. Their attitudes toward euthanasia are essential for shaping healthcare policies and training programs, as these professionals must navigate complex moral and practical considerations in their daily practice. Understanding these perspectives is key to ensuring appropriate policy development and providing effective education for healthcare providers.

**Objective:**

This study aims to analyze the sociodemographic factors influencing attitudes toward euthanasia among healthcare professionals in the Balearic Islands and examine the correlation between two scales measuring these attitudes.

**Methods:**

A cross-sectional study was conducted with a sample of 746 healthcare professionals from the Balearic Health Service. Participants completed a validated questionnaire assessing their attitudes toward euthanasia with the Euthanasia Attitude Scale and the Attitude Toward Euthanasia, along with a socio-demographic questionnaire.

**Results:**

The findings reveal that attitudes toward euthanasia are significantly influenced by variables such as age, gender, religious beliefs, and professional experience. Younger professionals, males, and those with fewer religious convictions tend to express more favorable attitudes toward euthanasia. Differences were also observed across professional categories, with nurses generally showing greater acceptance compared to physicians and other healthcare workers.

**Conclusion:**

Sociodemographic factors play a crucial role in shaping healthcare professionals’ attitudes toward euthanasia. These findings underscore the importance of tailored educational strategies and policy considerations to address diverse perspectives within the healthcare sector.

## Background

In the field of healthcare, ethics focuses on the study of moral principles that guide and assess the conduct of professionals in the care process [1, 2].

In end-of-life patient care, these ethical implications become even more prominent due to the patients’ heightened state of vulnerability [3, 4]. In this context, respecting patient autonomy by involving them in decisions affecting their care becomes even more essential [5–7]. Thus, the paradigm of patient-centered care should shift toward a more humanistic perspective, which includes, among other aspects, improved communication tools, a meaningful professional-patient relationship, and, in most cases, the integration of spirituality as a central axis of holistic care for these patients [8–11]. This humanistic approach provides both professionals and patients, as well as their families, with a more fulfilling experience and a significantly more positive perception of the end-of-life process [12].

However, when healthcare professionals face requests for Medical Assistance in Dying (MAID), the care team-patient relationship may be challenged, especially when the ethical assessment of the situation conflicts with the patient’s wishes [13, 14]. These ethical tensions often arise from a clash between the principle of respect for patient autonomy and the professional’s own moral or religious beliefs, as well as institutional norms. Attitudes toward euthanasia are therefore not merely individual opinions but reflect deeper ethical stances that shape decision-making in clinical settings. Professionals may experience moral distress when their ethical judgments are at odds with patient wishes, particularly in jurisdictions where euthanasia is legal. Understanding these ethical dimensions is crucial for supporting professionals in end-of-life care decisions that balance compassion, autonomy, and ethical responsibility [15]. The term MAID, which includes euthanasia practices and assisted suicide, generates bioethical dilemmas and is a source of controversy in the regulatory processes where it has been implemented across different socio-historical contexts. In Spain, the current legal framework regulates both euthanasia and assisted suicide under the umbrella of “medical assistance in dying.” Euthanasia is defined as the practice in which healthcare professionals directly administer life-ending interventions upon the patient’s voluntary and well-considered request. Assisted suicide involves the provision of the means for the patient to self-administer life-ending medication with medical support. Both practices are legally permitted, although assisted suicide is less commonly performed and less familiar among healthcare professionals in Spain. However, in some countries such as the Netherlands, Luxembourg, Canada, and certain states in the United States, assisted suicide is legally permitted and constitutes a distinct practice from euthanasia [16]. To avoid confusion, this study focuses specifically on attitudes toward euthanasia within the context of the Spanish healthcare system.

The study of attitudes traces its origins to the psychologist Herbert Spencer, who first introduced the term in 1862 [17]. After various debates in the field of social psychology, a consensus was reached in defining attitude as an “evaluative response that predisposes an individual to engage in a behavior either favorably or unfavorably” [18]. However, there is consensus that an attitude alone cannot predict specific behavior, although standardized measurement methods have been established based on attitude scales, such as the Thurstone and Likert attitude scales [19–21]. Understanding healthcare professionals’ attitudes toward euthanasia is essential beyond purely academic interest. These attitudes directly influence decision-making processes, patient care, and the ethical climate within healthcare settings. Moreover, knowledge of these attitudes provides valuable insights for shaping political strategies aimed at regulating end-of-life care and developing targeted training programs that prepare healthcare professionals to face ethical dilemmas in clinical practice. Thus, exploring the determinants of such attitudes can contribute to improving both policy-making and educational interventions in the healthcare sector.

**Fig. 1:**
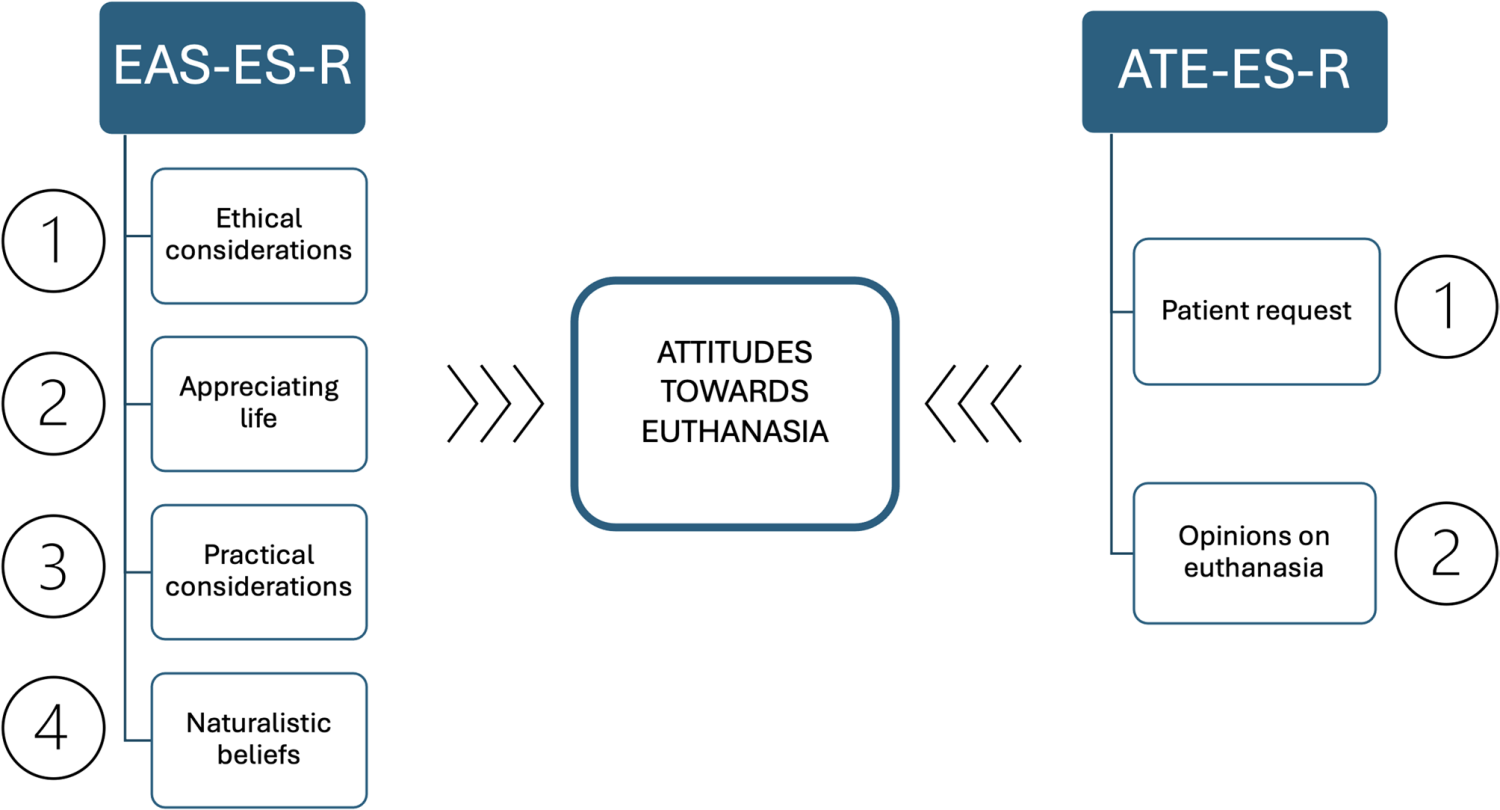
Dimensions of the EAS-ES-R and ATE-ES-R questionnaires that influence attitudes toward euthanasia

Regarding euthanasia, two measurement scales for these attitudes can be found: Euthanasia Attitudes Questionnaire (EAS) [22] and Euthanasia Attitudes Scale (ATE) [23]. Both scales contain various items that are answered based on Likert-type response alternatives and have been validated in different contexts and languages, demonstrating their validity for measuring respondents’ attitudes toward euthanasia [22–31] (See Fig. 1).

The debate on euthanasia has led to varying regulatory approaches across countries facing early legislative attempts in the United States date back to 1906, although these initiatives were ultimately unsuccessful [32]. Highly publicized cases, such as those of Karen Ann Quinlan and Paul Brophy, brought the issue into public and legal discourse, prompting broader societal reflection. Since then, regulatory frameworks have progressively emerged [33, 34]. The Northern Territory of Australia became the first jurisdiction to legalize euthanasia in 1996 [35, 36]. In the United States, Oregon legalized physician-assisted dying at the state level in 2005 [37], while Canada followed with nationwide legalization of medical assistance in dying (MAiD) in 2016 through amendments to its Criminal Code, following pivotal legal cases such as *Carter v. Canada* [38, 39]. Similarly, New Zealand legalized euthanasia for terminally ill patients following a parliamentary vote in 2019 and a national referendum in 2020 [40].

In Europe, the Netherlands pioneered the regulation of euthanasia with provisional legislation in 1994, which was fully implemented in 2002 [41]. Belgium followed that same year, with a swift legislative process due to pre-existing informal practices, particularly in Flanders [42]. Luxembourg enacted a similar law in 2008 alongside its palliative care legislation, allowing euthanasia and assisted suicide under strict conditions [43]. Spain became the fourth European country to legalize euthanasia and assisted suicide in 2021, followed by Portugal in 2023 [44, 45]. In Latin America, Colombia recognized the right to die with dignity in 1997, with national guidelines introduced in 2014 [46]. Most recently, Ecuador decriminalized euthanasia in 2024 following a Constitutional Court ruling in the case of Paola Roldán, a patient with amyotrophic lateral sclerosis (ALS) [47].

These legal developments reflect not only societal shifts but also ethical and professional challenges, particularly among healthcare providers who care for patients at the end of life. In this context, analyzing healthcare professionals’ sociodemographic characteristics in relation to their values and attitudes toward euthanasia is crucial. Such understanding can inform institutional policies and contribute to more ethically aligned and resource-sensitive healthcare practices.

Despite the existence of validated scales to measure healthcare professionals’ attitudes toward euthanasia, few studies have simultaneously applied both instruments in the same population to compare their performance and correlation. Furthermore, limited evidence is available regarding how sociodemographic factors influence these attitudes in specific regional contexts such as the Balearic Islands, where legislation on medical assistance in dying has recently evolved. Addressing these gaps can contribute to a better understanding of how personal and contextual factors shape professional positioning on end-of-life care practices.

Based on this background, the present study has two main objectives: [1] to assess the sociodemographic determinants influencing healthcare professionals’ attitudes toward euthanasia in the Balearic Islands, and [2] to evaluate the correlation of these attitudes across both validated scales (EAS and ATE). Accordingly, we hypothesize that: (H1) certain sociodemographic characteristics—such as age, sex, and religiosity—will be significantly associated with more favorable or unfavorable attitudes toward euthanasia; and (H2) both attitude scales will show a strong positive correlation, supporting their convergent validity in this professional context.

## Methodology

### Participants

The study aimed to involve healthcare professionals working in the Balearic Health Service. To recruit participants, approval was first obtained from the relevant management teams through their respective research committees. Following this, the questionnaire was distributed to approximately 4500 healthcare professionals through via a corporate email sent to all professionals, and a direct access link was provided on the intranet of each healthcare center. Data collection took place between July and November 2023, resulting in 746 completed responses, resulting in an estimated response rate of 16.6%. Due to the anonymous nature of the survey, data on explicit refusals or partial completions were not available. The average response time was 16:36 min for completing the sociodemographic questionnaire and the EAS-ES-R scale [48] and the ATE-ES-R scale [49].

The questionnaire was completed anonymously, and no personal data were recorded. Participation was voluntary, and prior to accessing the questionnaire, all participants were required to read an information sheet and give their informed consent by ticking a mandatory checkbox, in accordance with ethical approval protocols. The criteria for inclusion were as follows: the participant had to be a healthcare professional working within the Balearic Health System, have an understanding of the language and concepts used in the instrument, consent to participate in the study, and sign the informed consent form, granting permission for the use of the data for scientific research.

### Compliance with ethical standards

The research project proposal was approved by the Research Ethics Committee of the Balearic Islands, meeting the methodological, ethical, and legal requirements outlined in the 2013 Declaration of Helsinki under code CEI: IB 5116/23 PI. The study includes a Patient Information Sheet about the project and an Informed Consent Form. The data obtained are confidential and cannot be used for any purpose other than the objective of this study.

### Instruments

The questionnaire used for data collection consists, in addition to the Patient Information Sheet and the Informed Consent Form, of three main components: the sociodemographic questionnaire, the Revised Euthanasia Attitudes Questionnaire (EAS-ES-R), and the Revised Euthanasia Attitudes Scale (ATE-ES-R).

### Sociodemographic questionnaire

The sociodemographic questionnaire is divided into two main sections. The first part collects personal, professional, and work-related data, while the second part addresses the ethical implications involved in the provision of assisted dying.

The questionnaire gathers data on age, gender, marital status, whether the respondent has children, personal or family history of serious or incurable illnesses, and any recent loss of a significant family member. It also collects work experience details, such as years of experience, profession, specialization, workplace, any management positions held, and membership in healthcare or clinical ethics committees. Furthermore, it includes questions about the work shift, types of patients attended to, religious beliefs, knowledge of the euthanasia law, and any training received in ethics and euthanasia.

The questionnaire also examines attitudes toward euthanasia in the public healthcare system, conscientious objection, and whether palliative care could prevent the need for euthanasia. Additionally, it inquires about participation in the care of patients requesting assisted dying, covering various contexts such as receiving the request, participation in decision-making, medication administration, and other related aspects. Finally, it asks respondents to assess their satisfaction with their involvement in the care of patients requesting assisted dying and whether they required psychological support following the intervention.

### Euthanasia attitudes questionnaire EAS-ES-R

The scale consists of a revision of the EAS-ES scale proposed by Onieva-Zafra in 2022 [25], resulting in the EAS-ES-R instrument [48] (see Table 1), which is made up of 21 items, 4 of which have been revised and updated. Each item is rated using a 5-point Likert scale with responses ranging from 5 to 1, as follows: 5 = strongly agree, 4 = agree, 3 = neither agree nor disagree, 2 = disagree, and 1 = strongly disagree. The total score range of the scale varies from 21 to 105. Higher scores indicate more favorable positions toward euthanasia. The scale is structured into four factors: ethical considerations, appreciating life, practical considerations, and naturalistic beliefs. The questionnaire demonstrated high internal consistency, with a Cronbach’s alpha of 0.892.

**Table 1:** Escala EAS-ES-R

EAS-ES-*R* Scale	
Factor	Ítem
**Ethical considerations**	1.c. Euthanasia should be accepted in today’s society.
	1.k. Euthanasia gives a person a chance to die with dignity.
	1.e. Euthanasia is helpful at the right time and place.
	1.f. Euthanasia is a human act.
	1.g. Euthanasia should be illegal.
	2.d. I trust in the local medical system to implement euthanasia properly.
	1.a. A person with a terminal illness has the right to decide to die.
	1.d. There are no cases when euthanasia is appropriate.
	1.i. The taking of human life is wrong no matter what the circumstances.
	1.j. Euthanasia is acceptable in cases when all hope of recovery is gone.
	2.c. Euthanasia will lead to abuses.
**Appreciating life**	3.b. Euthanasia should be practiced only to eliminate physical pain and not emotional pain.
	3.d. One of the key professional ethics of physicians is to prolong lives, not to end lives.
	3.c. One’s job is to sustain and preserve life, not to end it.
	3.a. There are very few cases when euthanasia is acceptable.
**Practical considerations**	2.a. Euthanasia is acceptable if the person is old.
	2.b. If a terminally ill or injured person is increasing concerned about the burden that his/her deterioration of health has placed on his/her family, I will support his/her request for euthanasia.
	1.b. Inducing death for compassionate reason is wrong.
	1.h. Euthanasia should be used when the person has a terminal illness.
**Naturalistic beliefs**	4.a. A person should not be kept alive by machines.
	4.b. Natural death is the consolation for suffering.

### Attitude toward euthanasia ATE-ES-R

The scale is a revision of the ATE-ES scale by Fernández-Martínez [31] from 2020. The ATE-ES-R scale [49] (see Table 2) consists of 10 items, which are answered using a Likert scale ranging from 1 to 5, where: [1] strongly disagree [2], disagree [3], undecided [4], agree, and [5] strongly agree. The total score range of the scale is between 10 and 50. Higher scores indicate more favorable attitudes toward euthanasia. This scale is structured into two factors: in favor of euthanasia and patient request and demonstrated strong reliability, with a Cronbach’s alpha of α = 0.889.

**Table 2:** ATE-ES-R scale

ATE-ES-*R* Scale	
Factor	Ítem
**Patient request**	1.If a patient in severe pain requests it, the care team should remove life support and allow that patient to die.
	10.If a dying patient requests it, the care team should remove their life support and allow them to die.
	3.If a patient in severe pain requests it, the care team should prescribe that patient enough medicine to end their life.
	8.If a dying patient requests it, the care team should prescribe enough medicine to end their life.
**Opinions on euthanasia**	6.Even if the care team does not think that a patient will NR recover, it would be wrong for the care team to end the life of a patient.
	9.Even if the care team knows that a patient is in severe and uncontrollable pain, it would be wrong for the care team to end the life of that patient.
	5.It is okay for the care team to administer enough medicine to a suffering patient to end that patient’s life if the care team thinks that the patient’s pain is too severe.
	2.It is okay for the care team to administer enough medicine to end a patient’s life if the care team does not believe that they will recover.
	4.It is okay for the care team to remove life-support and let a patient die if the care team does not believe the patient will recover.
	7.It is okay for the care team to remove a patient’s life-support and let them die if the care team thinks that the patient’s pain is too severe.

*Both instruments used in this study (EAS-ES-R and ATE-ES-R) are validated Spanish versions adapted for the Spanish healthcare context* [48, 49]. *Permission to use the instruments for research purposes was obtained from the authors of the respective validated versions. A formal pre-test was not conducted; however*,* the instruments used were previously validated in the Spanish context and reviewed by experts*,* which helped to ensure content clarity.”*

### Statistical analysis

The statistical analysis was conducted using IBM SPSS AMOS version 26. Initially, data coding and exploration were performed, followed by the calculation of new variables according to the correction criteria established for the used questionnaires. A descriptive analysis was then carried out to assess the sample composition. For categorical variables, frequencies with percentages were calculated, while for continuous variables, means and standard deviations were determined. To test for normality in the quantitative variables, the Kolmogorov-Smirnov test (*N* > 50) was applied. The correlation and statistical significance between the sociodemographic variables and the scales were examined, as well as the correlation between attitudes measured by the EAS-ES-R and ATE-ES-R scales. Finally, Spearman’s correlation was applied, and a heatmap was generated using MetaboAnalisis version 5.0.

In addition, a multiple linear regression analysis was performed using the enter method to identify which variables independently predicted overall attitudes toward euthanasia and assisted suicide (EAS), while controlling for potential confounding factors. The following nine independent variables were included: sex, age, marital status, having children, having a professional specialization, years of professional experience, considering oneself a religious person, having received training on euthanasia, and having received training on ethics.

## Results

### Sample description

A response rate of 16.56% was obtained, with a total of 746 participating healthcare professionals (Table 3). The sample was predominantly female (75.7%), with an age range from 22 to 66 years (M = 43.56, SD = 10.46). Regarding marital status, 41.7% were single, 47.7% married, 9.5% separated or divorced, and 1.1% widowed. Concerning parenthood, 44.9% of participants had no children, while 55.1% did. Professional experience was relatively evenly distributed across different time ranges, with a slightly higher proportion between 21 and 30 years. The majority of respondents (61.7%) practiced in Mallorca, followed by Menorca (12.7%) and Ibiza-Formentera (25.6%). In terms of professional setting, 31.4% worked in primary care, 49.1% in specialized care, 2.0% in out-of-hospital emergency services, 9.1% in management positions, and 8.4% in other settings.

**Table 3:** Main sociodemographic variables included in the study

Variable	Category	*N*	%
Sex	Man	180	24.1
	Women	565	75.7
Country of birth	Spain	687	92.1
	Other EU country	17	23
	Non-EU country	42	5.6
Marital status	Single	311	41.7
	Married	356	47.7
	Separated/Divorced	71	9.5
	Widowed/widower	8	1.1
Do you have any children?	No	335	44.9
	Yes	411	55.1
Workplace	Mallorca	359	61.7
	Menorca	74	12.7
	Ibiza-Formentera	149	25.6
Type of health profession	University	581	77.9
	Non-university	165	22.1
Type of university profession	Nursing	360	59.2
	Medicine	167	27.5
	Pharmacy	7	1.2
	Physiotherapy	14	23
	Dentistry	4	0.7
	Social work	14	23
	Psychology	2	0.3
	Others	40	6.6
Specialty	No	335	44.9
	Yes	273	36.6
Scope of care	Specialized care	366	49.1
	Primary care	234	31.4
	Out-of-hospital emergency device	15	2
	Management position	68	9.1
	Others	63	8.4
Have a serious/incurable illness	No	722	96.8
	Yes	24	3.2
Having a family member with a serious illness	No	591	79.2
	Yes	155	20.8
Recent loss of a family member	No	466	62.5
	Yes	280	37.5
Consider yourself a religious person	No	555	74.4
	Yes	191	25.6

### Knowledge and ethical attitudes

42.9% of the sample reported having little to no knowledge about the assisted dying law. 50.4% (*N* = 376) stated they had received some form of ethics training, although only 22.9% considered themselves adequately trained in this area. Regarding specific training in this area 31.1% (*N* = 232) reported having received training, and only 12.3% of the total sample considered themselves adequately trained. Additionally, 4.6% of participants had been members of a clinical ethics committee, and 3.2% of a clinical research ethics committee. Concerning issues related to the professional context, 92.6% of participants supported the provision of euthanasia in the public health system, 93.8% stated that such provision does not compromise the essence of their profession, and 76.5% affirmed that palliative care does not prevent requests for euthanasia from patients. 12.6% of professionals identified as conscientious objectors to the provision of euthanasia, while 66.4% emphasized that the patient’s request should take priority over this objection. Finally, only 16.8% of professionals reported having participated in the care of patients who requested assisted dying. Among these, 36% participated in providing end-of-life support, while 64% were part of the responsible care team (See Table 4).

**Table 4:** Questions on ethical issues regarding euthanasia included in the study

Questions about ethical issues involved in MAID		*N*	%
Are you aware of the national law on assisted dying?	None	48	6.4
	A little	474	63.5
	A lot	178	23.9
	Completely	46	6.2
Have you attended any ethics training?	No	370	49.6
	Yes	376	50.4
Do you consider yourself sufficiently educated in ethics?	No	575	77.1
	Yes	171	22.9
Have you attended any training on euthanasia?	No	514	68.9
	Yes	232	31.1
Do you consider yourself sufficiently educated about euthanasia?	No	654	87.7
	Yes	92	12.3
Are you or have you been a member of a Healthcare Ethics Committee?	No	712	95.4
	Yes	34	4.6
Are you or have you been a member of a Clinical Research Ethics Committee?	No	722	96.8
	Yes	24	3.2
Do you agree with the provision of euthanasia in the public health system?	No	55	7.4
	Yes	691	92.6
Do you think that, as healthcare professionals, involvement in the euthanasia process distorts the meaning of the profession?	No	700	93.8
	Yes	46	6.2
Do you think adequate palliative care would prevent patients from having to request euthanasia?	No	571	76.5
	Yes	175	23.5
Do you think that a patient’s request for euthanasia should take precedence over a professional’s conscientious objection?	No	251	33.6
	Yes	495	66.4
Do you consider yourself a conscientious objector to euthanasia?	No	652	87.4
	Yes	94	12.6
Have you ever participated in caring for a patient who has requested assistance in dying?	No	621	83.2
	Yes	125	16.8
In what area of assisted dying have you been involved?	Part of the end-of-life support team	45	36.0
	Part of the team responsible for the patient	80	64.0

### Attitudes toward euthanasia: ATE scale

Responses to the items on the ATE questionnaire showed a general tendency to position themselves between indecision and agreement (Table 5). Items ATE1, ATE2, ATE3, and ATE4 had means between 3.3 and 3.4, suggesting a considerable proportion of participants with a neutral position. However, variability was observed in the responses, with standard deviations greater than 1.1. Item ATE6 showed one of the lowest means (2.65), indicating a higher proportion of respondents inclined to disagree, a pattern similar to that observed in ATE9 (M = 2.66). In contrast, items ATE8 and ATE10 recorded the highest means (3.78). (See Table 5).

**Table 5:** Frequencies, percentages, mean and standard deviation by ATE item

Item	Strongly disagree	Disagree	Undecided	Agree	Totally agree	M	DT
ATE1	48 (6.4)	110 (14.7)	218 (29.2)	249 (33.4)	121 (16.2)	3.38	1,115
ATE2	66 (8.8)	115 (15.4)	192 (25.7)	243 (32.6)	130 (17.4)	3.34	1,190
ATE3	58 (7.8)	109 (14.6)	206 (27.6)	259 (34.7)	114 (15.3)	3.35	1,138
ATE4	59 (7,9)	126 (16,9)	195 (26,1)	260 (34,9)	106 (14,2)	3,31	1,145
ATE5	65 (8,7)	160 (21,4)	215 (28,8)	204 (27,3)	102 (13,7)	3,16	1,166
ATE6	111 (14,9)	249 (33,4)	221 (29,6)	120 (16,1)	45 (6,0)	2,65	1,100
ATE7	57 (7,6)	194 (26,0)	229 (30,7)	186 (24,9)	80 (10,7)	3.05	1,115
ATE8	34 (4.6)	72 (9.7)	110 (14.7)	337 (45.2)	193 (25.9)	3.78	1,075
ATE9	115 (15.4)	253 (33.9)	198 (26.5)	134 (18.0)	46 (6.2)	2.66	1,125
ATE10	26 (3.5)	70 (8.0)	109 (14.6)	348 (46.6)	203 (27.2)	3.78	1,075

### Attitudes toward euthanasia: EAS scale

Responses to the items on the EAS questionnaire indicated a high level of agreement in items 1.a, 1.c, 1.e, and 1.k, with means between 4.28 and 4.48 (Table 6). Item 1.g had the highest mean (4.53). In contrast, some items such as 2.a, 2.c, 3.a, 3.b, and 3.c showed lower values, between 2.5 and 3.5. Items 4.a and 4.b exhibited means in an intermediate range (3.3–3.5). (See Table 6)

**Table 6:** Frequencies, percentages, mean and standard deviation by EAS item

Item	Totally disagree	Disagree	Undecided	Agree	Totally agree	M	DT
1.a	23 (3.1)	12 (1.6)	25 (3,4)	209 (28.0)	477 (63.9)	4.48	,886
1.b	107 (14.3)	214 (28.7)	191 (25.6)	146 (19.6)	88 (11.8)	3.14	1,229
1.c	17 (2,3)	18 (2.4)	45 (6.0)	235 (31.5)	431 (57.8)	4,40	,883
1.d	335 (44,9)	265 (35,5)	95 (12,7)	24 (3,2)	27 (3,6)	4,15	1,005
1.e	17 (2,3)	20 (2,7)	35 (4,7)	275 (36,9)	399 (53,5)	4,37	,872
1.f	17 (2,3)	27 (3,6)	56 (7,5)	278 (37,3)	368 (49,3)	4,28	,918
1.g	503 (67,4)	187 (25,1)	24 (3,2)	11 (1,5)	21 (2,8)	4,53	,861
1.h	36 (4,8)	150 (20,1)	173 (23,2)	247 (33,1)	140 (18,8)	3,41	1,145
1.i	303 (40,6)	305 (40,9)	64 (8,6)	32 (4,3)	42 (5,6)	4,07	1,081
1.j	22 (2,9)	64 (8,6)	92 (12,3)	349 (46,8)	219 (29,4)	3,91	1,009
1.k	15 (2,0)	22 (2,9)	31 (4,2)	227 (30,4)	451 (60,5)	4,44	,866
2.a	115 (15,4)	302 (40,5)	163 (21,8)	111 (14,9)	55 (7,4)	2,58	1,137
2.b	42 (5,6)	131 (17,6)	199 (26,7)	255 (34,2)	119 (16,0)	3,37	1,115
2.c	181 (24,3)	278 (37,3)	197 (26,4)	68 (9,1)	22 (2,9)	3,71	1,026
2.d	22 (2,9)	31 (4,2)	83 (11,1)	343 (46,0)	267 (35,8)	4,08	,947
3.a	147 (19,7)	288 (38,6)	162 (21,7)	118 (15,8)	31 (4,2)	3,54	1,100
3.b	208 (27,9)	341 (45,7)	140 (18,8)	36 (4,8)	21 (2,8)	3,91	,952
3.c	229 (30,7)	351 (47,1)	58 (7,8)	63 (8,4)	45 (6,0)	3,88	1,119
3.d	208 (27,9)	362 (48,5)	80 (10,7)	62 (8,3)	34 (4,6)	3,87	1,055
4.a	29 (3,9)	161 (21,6)	225 (30,2)	224 (30,0)	107 (14,3)	3,29	1,077
4.b	132 (17,7)	284 (38,1)	193 (25,9)	93 (12,5)	44 (5,9)	3,49	1,099

### Associations between sociodemographic variables and attitudes toward euthanasia

The study investigated the associations between various sociodemographic variables and attitudes toward euthanasia, as measured by the ATE and EAS questionnaires and their respective dimensions. The results of these analyses are summarized in Table 7, with a heat map providing a visual representation of the strength and direction of these associations.

#### Religiosity

**Fig. 2:**
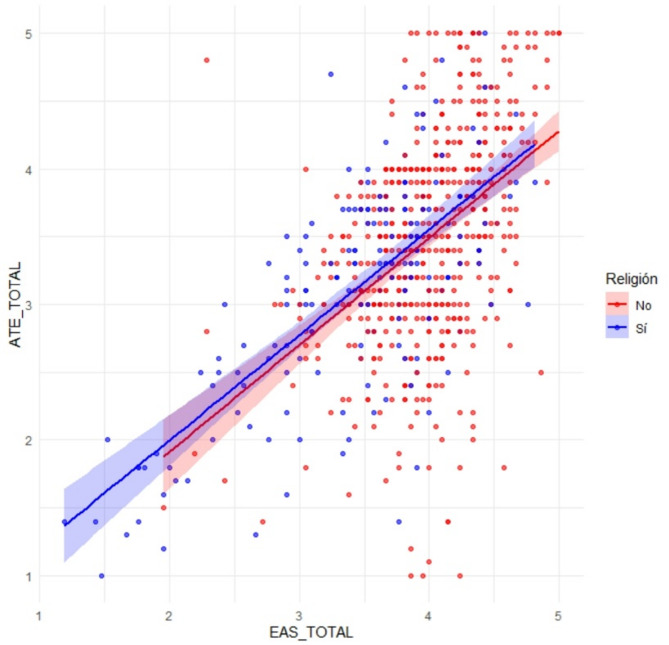
Religiosity and EAS/ATE Scores

The Mann-Whitney U test revealed statistically significant differences based on religious affiliation. On both the total EAS and ATE scales, individuals who do not identify as religious scored significantly higher than those who do (see Fig. 2), indicating more favorable attitudes toward euthanasia. Further analysis of the ATE subscales revealed that non-religious individuals also scored significantly higher on both ATE 1 and ATE 2 compared to religious individuals. Similarly, significant differences were found on the EAS subscales, specifically EAS 1, EAS 2, and EAS 3. These findings align with previous research suggesting that religious beliefs often play a significant role in shaping attitudes toward end-of-life decisions, with higher levels of religiosity generally associated with less favorable views on euthanasia.

#### Marital status

Significant differences were found in attitudes toward euthanasia based on marital status. Specifically, single individuals tended to express more favorable attitudes toward euthanasia compared to married individuals. This is supported by statistically significant differences in the total EAS scale (*p* = 0.001), where single participants exhibited a higher mean score (M = 3.9392, SD = 0.48921) compared to married participants (M = 3.7727, SD = 0.63421). Further analysis revealed significant differences in EAS 1 (*p* = 0.002) and EAS 2 (*p* = 0.020), with single participants scoring higher on both subscales (EAS 1: M = 4.3273, SD = 0.54783; EAS 2: M = 3.7248, SD = 0.69496) compared to married participants (EAS 1: M = 4.1309, SD = 0.77263; EAS 2: M = 3.5365, SD = 0.81915). These differences may reflect variations in social support, personal values, or life experiences that influence attitudes toward end-of-life choices.

#### Parenthood

Significant differences were found in attitudes toward euthanasia based on parenthood. Specifically, participants without children tended to express more favorable attitudes toward euthanasia compared to those with children. This is supported by statistically significant differences in the total EAS scale (*p* = 0.000), where participants without children exhibited a higher mean score (M = 3.9183, SD = 0.58340) compared to those with children (M = 3.7982, SD = 0.55760). Further analysis revealed significant differences in EAS1 (*p* = 0.000) and EAS2 (*p* = 0.020), with participants without children scoring higher on both subscales (EAS1: M = 4.2901,SD = 0.68490; EAS2: M = 3.7009,SD = 0.76509) compared to those with children (EAS1: M = 4.1620,SD = 0.67345; EAS2: M = 3.5674,SD = 0.75831).

#### Profession

Significant differences were observed between healthcare professions, with nursing professionals exhibiting more favorable attitudes toward euthanasia compared to physicians. This aligns with statistically significant differences found in most dimensions evaluated (*p* < 0.05), except for ATE 1 and EAS 4. Conversely, those without a specific healthcare specialty tended to be more favorable toward euthanasia than those with a specialty, a difference that was statistically significant across all dimensions analyzed (*p* < 0.05), including the total scale scores. These differences may be attributed to variations in training, roles, and experiences within the healthcare system. Nurses often have more direct and prolonged contact with patients at the end of life, which may shape their attitudes toward euthanasia.

#### Age and years of professional experience

Age and Years of Professional Experience: While both age and years of professional experience showed some associations with dimensions of the ATE and EAS scales (Table 7), further analysis revealed significant differences in the EAS score based on age groups (H = 23.713; *p* = 0.000). Specifically, the 28–37 age group tended to show a greater acceptance of euthanasia, with a gradual decrease in acceptance observed with increasing age. Differences in the EAS 1 factor were also significant between age groups (H = 25.430, *p* = 0.000), with the 28–37 age group displaying the highest scores. Similarly, the 28–37 age group showed the highest score in the EAS 2 factor. However, no significant differences were found in the ATE score or its dimensions based on age. Further analysis is needed to fully understand the nature of the relationships between age, experience, and attitudes toward euthanasia, as they may reflect cohort effects, changes in attitudes over time, or the impact of specific experiences in professional practice.

#### Experiences with seriously ill or deceased family members

The scores obtained among respondents who have had seriously ill family members or have experienced the loss of family members are generally not significant, with the exception of EAS 2 in both contexts. Those without these experiences scored higher on this subscale (*p* = 0.019 for having seriously ill family members and *p* = 0.010 for experiencing family loss). Additionally, statistically significant differences were found in the total EAS score in the context of the loss of a loved one, with those who have not experienced a family loss obtaining a higher score on the scale.

#### Knowledge of euthanasia law, ethics training, and euthanasia training

Knowledge of euthanasia law was associated with differences in attitudes, particularly in relation to ethical considerations. Specifically, significant differences were found in EAS 1 (H = 13.470; *p* = 0.004), with those having complete knowledge of the law presenting the lowest scores. Significant differences were also found in EAS 2 (H = 11.957; *p* = 0.008). No significant differences were found in EAS 3 (H = 3.478; *p* = 0.324) or EAS 4 (H = 3.169; *p* = 0.366).

Regarding the perception of ethical training, participants who did not believe they had sufficient ethical training obtained significantly higher scores on EAS 3 (M = 3.6896, SD = 0.73766) compared to those who considered their training sufficient (M = 3.4795, SD = 0.85982; U = 41563.5, *p* = 0.002).

Participants without euthanasia training obtained significantly higher scores on the total ATE scale (M = 3.3097, SD = 0.57425) compared to those who had received training (M = 3.1302, SD = 0.66453; U = 49705.5, *p* < 0.001). This trend was also observed in the ATE 1 (U = 48266.0, *p* < 0.001) and ATE 2 (U = 53074.5, *p* = 0.016) subscales. Conversely, no statistically significant differences were found in the total EAS scale or its dimensions (*p* > 0.05). Similarly, participants who did not believe they had sufficient euthanasia training obtained significantly higher scores on the ATE Total scale (M = 3.2749, SD = 0.58324; U = 26153, *p* = 0.042) and ATE 1 subscale (M = 3.6288, SD = 0.85195 U = 25630, *p* = 0.020) compared to those who believed they had sufficient training (ATE Total scale: M = 3.1043, SD = 0.75490; ATE 1 subscale: M = 3.3451, SD = 1.12039). There was no statistical significance found in any of the dimensions of the EAS or in the EAS total.

This suggests that familiarity with the legal and ethical framework surrounding euthanasia and the receipt of specific training may influence healthcare professionals’ attitudes and beliefs about end-of-life decisions, with targeted education playing a crucial role in fostering informed and nuanced perspectives on euthanasia.

**Table 7:** Kruskal-Wallis and Mann-Whitney tests for significant variables related to EAS and ATE

Variable	Element	Category	M	DT	U/H	*P*
**Religiosity**	**Total EAS**	No	3,9812	,43,799	29735,000	0.000**
		Yes	3,4769	,73,055		
	**Total ATE**	No	3,3105	,57,282	43641,500	0.000**
		Yes	3,0895	,67,908		
	**ATE 1**	No	3,7068	,82,884	38,743,000	0,000**
		Yes	3,2657	99,062		
	**ATE 2**	No	3,3243	,87,817	44,182,500	0.001**
		Yes	3,0681	,80,060		
	**EAS 1**	No	4,3703	48,430	30,851,500	0,000**
		Yes	3,7817	93,608		
	**EAS 3**	No	3,7441	,75,305	37,281,000	0,000**
		Yes	3,3429	,74,969		
	**EAS 2**	No	3,7827	66,527	31139,000	0,000**
		Yes	3,1759	84,930		
**Marital state**	**EAS total**	Single	3,9392	48,921	47174,500	0.001**
		Married	3,7727	63,421		
		Married	3,2551	,90,878		
	**EAS 1**	Single	4,3273	,54,783	47762,000	0.002**
		Married	4,1309	,77,263		
	**EAS 2**	Single	3,7248	,69,496	49595,500	0.020*
		Married	3,5365	,81,915		
**Children**	**Total EAS**	No	3,9183	,58,340	57855,000	0.000**
		Yes	3,7982	,55,760		
	**EAS 1**	No	4,2901	,68,490	57,898,000	0,000**
		Yes	4,1620	67,345		
	**EAS 2**	No	3,7009	,76,509	62059,500	0.020*
		Yes	3,5674	,75,831		
**Profession**	**EAS total**	Nurse	3,9275	53,782	24,590,500	0.001**
		Doctor	3,7160	70,050		
	**ATE total**	Nurse	3,2706	,59,132	26,119,000	0.015*
		Doctor	3,1293	,63,704		
	**ATE 1**	Nurse	3,6424	,86,946	24842,000	0.001**
		Doctor	3,3862	,92,273		
	**EAS 1**	Nurse	4,2950	,65,905	23703,500	0.000**
		Doctor	4,0198	,83,610		
	**EAS 3**	Nurse	3,6875	,79,534	26209,000	0.016*
		Doctor	3,5359	,73,133		
	**EAS 2**	Nurse	3,7611	,70,164	26,270,000	0.019*
		Doctor	3,5449	,86,836		
		Yes	3,2985	,67,633		
**Professional practice time**	**EAS 1**	0–5 years	4,2314	,74,733	13,076	0.023*
		5–10 years	4,3009	,61,277		
		11–15 years	4,2980	,62,931		
		16–20 years old	4,3080	,50,656		
		21–30 years old	4,1621	,72,988		
		> 30 years	4.0455	,76,161		
**Age**	**Total EAS**	18–27	3,8970	,56,043	23,713	0.000**
		28–37	3.9765	,55,075		
		38–47	3,8716	,51,930		
		48–57	3,7869	,59,121		
		> 58	3,6684	,66,473		
		48–57	3,2426	,85,697		
		> 58	3,2922	,94,264		
	**EAS 1**	18–27	4,2818	,69,338	25,430	0,000**
		28–37	4,3517	,67,906		
		38–47	4,2502	,57,618		
		48–57	4,1421	,72,754		
		> 58	4,0049	,77,426		
	**EAS 2**	18–27	3,5927	,68,092	23,384	0,000**
		28–37	3,8103	,68,079		
		38–47	3,6823	,74,506		
		48–57	3,5446	,78,449		
		> 58	3.3235	,87,953		
**Family member with illness**	**EAS 2**	No	3,6575	,75,944	40229,500	0.019**
		Yes	3,5123	,77,160		
**Loss of a family member**	**Total EAS**	No	3,9022	,49,968	59068,500	0.030*
		Yes	3,7687	,66,826		
	**EAS 2**	No	3,6979	,70,013	57957,000	0.010*
		Yes	3,5100	,84,762		
**Knowledge of the euthanasia law**	**Total EAS**	None	3,9058	,53,249	14,675	0.002**
		A little	3.8238	,51,275		
		A lot	3.9489	,60,685		
		Completely	3,7133	,91,743		
	**Total ATE**	None	3,3979	,50,212	15,494	0,001**
		Alittle	3,2759	,57,279		
		A lot	3,2725	,60,560		
		Completely	2,8043	,86,666		
	**ATE 1**	None	3,9219	,74,226	20,791	0,000**
		A little	3,6108	,82,195		
		A lot	3,6362	,90,980		
		Completely	2,9130	1,29767		
	**ATE 2**	None	3,5938	,89,344	19,502	0,000**
		A little	3,2431	,74,294		
		A lot	3,3172	,78,853		
		Completely	3,2753	,94,134		
	**EAS 1**	None	4,3083	,59,888	13,470	0,004**
		A little	4,1947	,61,358		
		A lot	4,3219	,70,119		
		Completely	3,9870	1,14185		
	**EAS 2**	None	3,6208	,74,060	11,957	0.008**
		A little	3,5835	,71,171		
		A lot	3,7506	,80,859		
		Completely	3,6087	1.05237		
**Received** **ethical training**	**EAS 3**	No	3,6896	,73,766	41563,500	0.002**
		Yes	3,4795	,85,982		
	**Total ATE**	No	3,3097	,57,425	49705,500	0.000**
		Yes	3,1302	,66,453		
	**ATE 1**	No	3,6863	,84,015	48,266,000	0,000**
		Yes	3,3890	97,247		
	**ATE 2**	No	3,3149	,80,725	53074,500	0.016*
		Yes	3,1343	97,333		
	**ATE total**	No	3,2749	58,324	26,153,000	0.042*
		Yes	3,1043	75,490		
	**ATE 1**	No	3,6288	,85,195	25,630,000	0.020*
		Yes	3,3451	1.12039		

* The correlation is significant at the 0.05 level

** The correlation is significant at the 0.01 level

### Multiple linear regression analysis

#### Attitude toward euthanasia (ATE)

In total ATE, F = 4.118; *p* < 0.001, the model explained 4.4% of the variance in ATE scores. The Durbin-Watson statistic was 1.349, indicating no substantial autocorrelation of the residuals. See Table 8.

Two variables emerged as significant predictors. Identifying as a religious person was negatively associated with attitudes toward euthanasia (B = − 0.356; β = −0.191; *p* < 0.001), indicating that individuals who consider themselves religious tend to show lower acceptance of euthanasia. Having a professional specialization also showed a negative association (B = − 0.181; β = −0.109; *p* = 0.006), suggesting that professionals with a specialty tend to hold slightly less favorable attitudes toward euthanasia compared to those without a specialization.

#### Attitudes toward euthanasia and assisted suicide (EAS)

The model was statistically significant: F = 5.953; *p* < 0.001, with an adjusted coefficient of determination of R²adjusted = 0.069, indicating that approximately 6.9% of the variance in total EAS scores can be explained by the variables included in the model. The Durbin-Watson statistic was 0.155, suggesting possible positive autocorrelation of the residuals. See Table 9.

Among the included predictors, three were statistically significant. Having a professional specialization was negatively associated with attitudes toward euthanasia (B = − 0.210; β = −0.176; *p* < 0.001), indicating that professionals with a specialty had lower total EAS scores—that is, a slightly less favorable attitude toward this practice. Identifying as a religious person also showed a negative association (B = − 0.174; β = −0.130; *p* = 0.001), suggesting that those who consider themselves religious tend to show lower acceptance. Years of professional experience were positively associated with attitudes toward euthanasia (B = 0.040; β = 0.112; *p* = 0.020), indicating that greater experience is linked to a more favorable attitude toward this intervention.

Other variables—such as sex (*p* = 0.516), age (*p* = 0.038), marital status (*p* = 0.083), having children (*p* = 0.451), and having attended training on euthanasia (*p* = 0.228) or on ethics (*p* = 0.796)—did not show significant associations with the dependent variable.

**Table 8:** Multiple Linear Regression Predicting Attitudes Toward Euthanasia (ATE)

Predictor				B	SE	β	t	*p*	95% CI for B							
(Constant)				3.314	0.128	—	27.362	< 0.001	[3.242, 3.743]							
Professional specialty (yes = 1)				-0.181	0.066	-0.109	-2.731	0.006	[-0.311, -0.051]							
Religious (yes = 1)				-0.356	0.076	-0.191	-4.707	0.000	[-0.505 -0.208]							
Age (years)				0.001	0.001	0.046	1.085	0.279	[-0.001, 0.004]							
Sex (female = 1)				0.009	0.078	0.005	0.117	0.907	[-0.143, 0.162]							
Marital status (partnered = 1)				0.108	0.061	0.089	1.758	0.079	[-0.013, 0.228]							
Having children (yes = 1)				-0.051	0.087	-0.031	-0.593	0.553	[-0.222, 0.119]							
Years of professional experience				-0.09	0.024	-0.018	-0.371	0.711	[-0.056, 0.038]							
Training on euthanasia				0.058	0.077	0.032	0.745	0.457	[-0.094, 0.209]							
Training on ethics				0.078	0.072	0.047	1.095	0.274	[-0.062, 0.219]							

Note: B = Unstandardized coefficient; SE = Standard Error; β = Standardized coefficient (Beta); CI = Confidence Interval; Dependent variable: Total ATE score

Model summary: *R* = 0.260; R² = 0.067; Adjusted R² = 0.056; Durbin-Watson = 0.133; F(7, 770) = 6.171; *p* < 0.001

**Table 9:** Multiple Linear Regression Predicting Attitudes Toward Euthanasia (EAS)

Predictor				B	SE	β	t	*p*	95% CI for B							
(Constant)				3.660	0.97	—	37.5600	< 0.001	[3.468, 3.851]							
Professional specialty (yes = 1)				-0.210	0.47	-0.176	-4.486	0.000	[-0.302, -0.118]							
Religious (yes = 1)				-0.174	0.054	-0.130	-3.256	< 0.001	[-0.280, -0.069]							
Age (years)				0.002	0.001	0.087	2.081	0.038	[-0.000, 0.004]							
Sex (female = 1)				0.036	0.055	0.026	0.650	0.516	[-0.072, 0.144]							
Marital status (partnered = 1)				0.075	0.043	0.087	1.739	0.083	[-0.010, 0.161]							
Having children (yes = 1)				-0.046	0.061	-0.039	-0.754	0.451	[-0.167, 0.074]							
Years of professional experience				0.040	0.017	0.112	2.338	0.020	[-0.006, 0.074]							
Training on euthanasia				-0.66	0.055	-0.51	-1.206	0.228	[-0.173, 0.041]							
Training on ethics				0.013	0,051	0.011	0.259	0.796	[-0.86, 0.113]							

Note: B = Unstandardized coefficient; SE = Standard Error; β = Standardized coefficient (Beta); CI = Confidence Interval; Dependent variable: Total EAS score

Model summary: *R* = 0.297; R² = 0.088; Adjusted R² = 0.077; Durbin-Watson = 0.605; F(7, 770) = 8.256; *p* < 0.001

### Correlation between the ATE and EAS scales

**Fig. 3:**
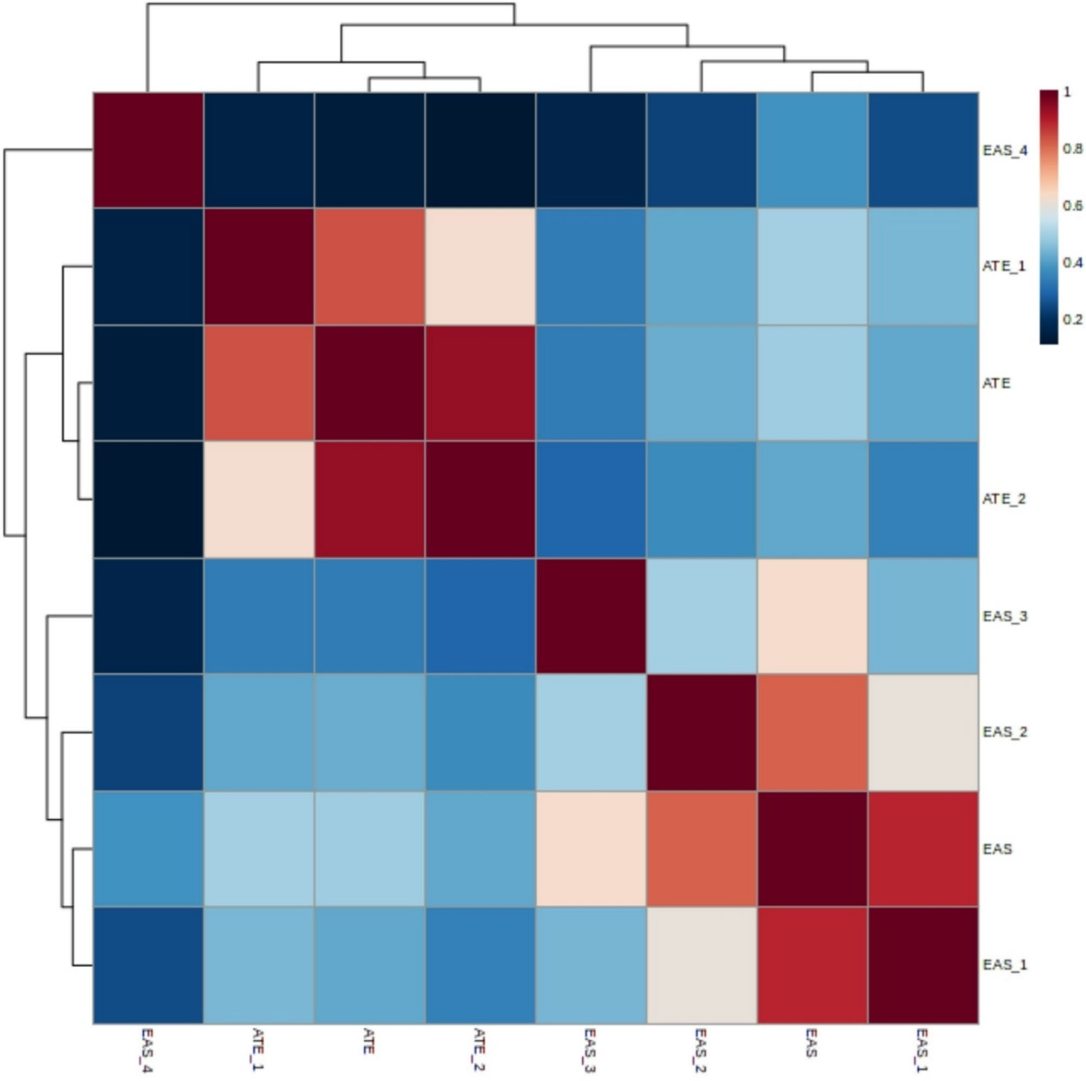
Heat Map of Correlations Between EAS and ATE Scales and Dimensions

The relationship between attitudes toward euthanasia as measured by the ATE and EAS scales was examined using Spearman’s rank-order correlation. This analysis revealed a statistically significant and positive correlation of moderate magnitude between the total scores on the ATE and EAS scales (*r* = 0.491, *p* < 0.001). This finding indicates that there is a tendency for participants with more favorable attitudes toward euthanasia as measured by one scale to also exhibit more favorable attitudes as measured by the other. However, the moderate correlation suggests that while the two scales are related, they are not measuring the exact same constructs, and there are other factors influencing attitudes toward euthanasia that are not captured by these scales. See Fig. 3.

In addition to the correlations between the total scores and ATE dimensions, relationships between the specific dimensions of the ATE and EAS scales were also examined. These analyses identified significant relationships of varying magnitudes (low to moderate) between specific dimensions of both scales, providing a more comprehensive understanding of the relationships between different facets of attitudes toward euthanasia. Detailed results of these analyses are presented in Table 10. All mentioned correlations were significant at the 0.01 level (two-tailed).

The observed correlations suggest that while the ATE and EAS scales both measure attitudes toward euthanasia, they capture different aspects of this complex construct. The EAS appears to reflect a more general attitude, while the ATE provides a more detailed assessment of specific ethical and practical considerations. The significant inter-scale correlations support the validity of both measures in assessing attitudes toward euthanasia among healthcare professionals.

**Table 10:** Correlations between the total EAS questionnaire and ATE scores and their dimensions

		EAS total	ATE total	EAS 1	EAS 3	EAS 2	EAS 4	ATE 1	ATE 2
**EAS total**	r	1,000	,491^**^	,894^**^	,633^**^	,819^**^	,381^**^	,497^**^	,419^**^
	*p*	.	,000	,000	,000	,000	,000	,000	,000
**ATE total**	r	,491^**^	1,000	,416^**^	,340^**^	,427^**^	,139^**^	,839^**^	,943^**^
	*p*	,000	.	,000	,000	,000	,000	,000	,000
**EAS 1**	r	,894^**^	,416^**^	1,000	,442^**^	,603^**^	,252^**^	,447^**^	,345^**^
	*p*	,000	,000	.	,000	,000	,000	,000	,000
**EAS 3**	r	,633^**^	,340^**^	,442^**^	1,000	,496^**^	,166^**^	,337^**^	,295^**^
	*p*	,000	,000	,000	.	,000	,000	,000	,000
**EAS 2**	r	,819^**^	,427^**^	,603^**^	,496^**^	1,000	,233^**^	,418^**^	,367^**^
	*p*	,000	,000	,000	,000	.	,000	,000	,000
**EAS 4**	r	,381^**^	,139^**^	,252^**^	,166^**^	,233^**^	1,000	,151^**^	,115^**^
	*p*	,000	,000	,000	,000	,000	.	,000	,002

** The correlation is significant at the 0.01 level (two-tailed)

## Discussion

The results obtained indicate that the variable religiosity plays the most significant role as a mediator in attitudes toward euthanasia. Specifically, individuals who identify as religious show a significantly higher rejection of euthanasia, which is reflected in both the overall scores of the questionnaires and in all of their dimensions, except for Factor 4 of the EAS questionnaire, labeled “naturalist beliefs.” This factor includes statements such as “A person should not be kept alive by machines” and “Natural death is the consolation for suffering,” which do not, in themselves, imply a clear stance for or against euthanasia. Rather, these statements reflect a view aligned with the acceptance of natural death, without medical intervention to artificially prolong life.

This finding is reflected in studies such as that of Terkamo-Moisio et al. [50] in 2019, which surveyed postgraduate nurses and found that identifying as religious had a negative impact on attitudes toward their role in euthanasia care, indicating they were less prepared to provide care in this context. Arreciado et al. [51] in 2024 also linked the lack of religious identity to more favorable attitudes toward euthanasia in nursing students. The predictive value of the variable “being religious” in showing different attitudes toward euthanasia is also demonstrated in other previous studies [52–59].

These findings can also be interpreted through broader theoretical frameworks. Religiosity, for instance, has been consistently linked to deontological moral reasoning and a stronger adherence to absolute moral norms, which may conflict with the principles underpinning the acceptance of euthanasia. From this perspective, religious individuals may be more inclined to reject euthanasia on the basis of the sanctity of life, regardless of individual autonomy or perceived suffering.

Regarding gender, no significant differences were found in attitudes towards euthanasia, and it was not a significant predictor in the regression model either. Although previous studies [60–63] have shown that males tend to exhibit more favorable attitudes toward euthanasia, the results of this study align with other studies [52, 64] that do not show this trend, likely due to the fact that the majority of respondents were women. This result is likely due to the fact that most respondents were women, which may have introduced a gender bias.

When considering these three variables—marital status, parenthood, and age—it is notable that the ATE questionnaire did not identify them as significant in relation to attitudes toward euthanasia, whereas the EAS did. This suggests that, while both instruments measure overall attitudes toward euthanasia in a similar direction, their psychometric properties differ, displaying varying sensitivities to specific variables.

In terms of marital status, being single was associated with a higher degree of agreement on euthanasia across the entire EAS questionnaire and in its Factors 1 and 2. This finding aligns with the study by Hawrelack et al. [65] where single individuals exhibited more favorable attitudes toward assisted dying, both in terminal illness situations and in cases of mental illness. This trend is also reflected in the regression models for EAS Factor 3 and ATE1, where being single is linked to more positive attitudes toward euthanasia. This may be explained by the particular relevance that the principle of autonomy holds for individuals without parental ties, as it contributes to a greater sense of control over one’s own life and a heightened awareness of personal quality of life. This is consistent with data from countries such as Belgium, the Netherlands, and the state of Oregon, where most requests for medical assistance in dying come from single individuals [66].

Not having children was also significantly associated with more favorable attitudes toward euthanasia in the EAS questionnaire and in its Factors 1 and 2. This finding is consistent with the study by Lachowski et al. [59] which reported that support for euthanasia decreases as family size increases. Regarding age, respondents aged 28 to 37 showed significantly greater acceptance of euthanasia across the entire EAS questionnaire and in its Factors 1 and 2. Additionally, there was a clear trend indicating that agreement with euthanasia decreased as age increased, aligning with the findings of Shekhawat et al. [67]. In the regression model, however, this variable was not significant in either questionnaire, suggesting the presence of confounding variables related to having offspring—such as age or marital status—which did show significance in the analysis.

Regarding age, respondents between 28 and 37 years old showed significantly greater acceptance of euthanasia across the entire EAS questionnaire and in its Factors 1 and 2. Additionally, there was a clear trend indicating that agreement with euthanasia decreased as age increased, consistent with the findings of Shekhawat et al. [67]. The regression analysis supports this pattern in Factor 3 of the EAS, where younger age emerged as a predictor of more positive attitudes towards euthanasia. However, it should be noted that age is not a strong predictor, as the literature reviewed reports mixed results. The practical reasoning behind ending one’s own life based on personal autonomy at younger ages may underlie this outcome. Age, although showing only a modest effect, could reflect generational shifts in moral socialization or increased professional exposure to complex end-of-life situations. Such exposure might foster a more pragmatic or experience-based approach to ethical decision-making, leading to slightly more favorable attitudes among older professionals [68].

A comparison of attitudes between physicians and nurses shows that nurses hold a more favorable view of euthanasia than physicians, which is consistent with previous studies such as that of Light et al. [69]. However, other studies, such as that of Stergiannis [70], did not find significant differences between these professions. The regression model, however, does not identify significant differences based on profession. In this case, variables such as gender or level of specialization may act as confounding factors. Moreover, the fact that the Spanish Law regulating euthanasia designates the ‘responsible physician’ and the ‘consulting physician’ as key figures in the process may influence more reflective attitudes and greater bioethical debate among these healthcare professionals [44].

The analysis of attitudes in relation to years of professional experience shows that, in the ‘ethical considerations’ factor, having fewer years of experience is significantly associated with a more favorable view of euthanasia. This trend is also reflected in the study by Bachmetjev et al. [57] in the context of palliative care, as well as in the study by Ortega Galán et al. [71] with nurses working in the Spanish public healthcare system. In the regression model, however, this variable reverses when other relevant variables are added. Thus, in the analysis of the total EAS score and EAS factors 1 and 2, more years of professional experience are associated with more favorable attitudes towards euthanasia. This discrepancy suggests the presence of confounding variables such as age or level of specialization, which may influence the direction of the association. These findings highlight the importance of conducting bivariate analyses to better capture the complexity of the factors associated with attitudes towards euthanasia.

When considering whether participants have a medical specialty, the data show that those without a specific healthcare specialization tend to hold more favorable attitudes toward euthanasia compared to professionals with a specialty. This trend is reflected across all dimensions analyzed, including the total scores of the scales. In the regression model, we observe a similar result, except in Factor 3 of the EAS, where this variable is not significant. These findings suggest that a higher level of specialization is associated with a more conservative view on euthanasia. However, the complexity of this variable is highlighted in studies such as that by Dopelt et al. (2021), where attitudes are mediated by the type of specialty, with internal medicine specialists being the most supportive of euthanasia [16]. Furthermore, less professional experience often implies less specialization and younger age, which is also evident in the association analysis, where professionals without a specialty tend to show more favorable attitudes toward euthanasia.

Having a close relationship with someone suffering from a serious or incurable illness, or having recently experienced the loss of a significant person, was only positively associated with Factor 2 of the EAS (appreciation of life). This finding highlights healthcare professionals’ preference for facilitating a ‘dignified death’ as a service that emphasizes life without suffering and respects patient autonomy [72, 73].

Differences in attitudes according to the level of knowledge about the assisted dying law were not significant in the overall questionnaire. However, greater knowledge of the law was associated with more negative attitudes toward euthanasia in EAS Factor 1 (ethical considerations). In contrast, the regression model did not identify this variable as a predictor of attitudes, which suggests that the level of knowledge about the law may, in turn, be mediated by other variables. This finding underscores how ethical values are influenced by individual morality and suggests that professionals with more negative attitudes toward euthanasia tend to be more engaged in understanding the legal framework that regulates these practices in a specific context [74].

Regarding professionals who have received prior training on euthanasia and their perception of being well-trained in this area, the ATE questionnaire identifies a relationship between a lack of training or insufficient preparation in euthanasia and more favorable attitudes toward it. However, due to its different psychometric properties, the EAS questionnaire does not detect this association. The tendency to exhibit more positive attitudes toward euthanasia despite perceiving oneself as inadequately trained may be explained by the fact that attitudes and values regarding euthanasia are often shaped before professional training and may remain unchanged even after receiving formal education on the subject [75, 76].

With respect to euthanasia training, the study by Testoni et al. [55] highlights the significance of this variable, as it reveals that assisted dying is often mistaken for other end-of-life care practices. This pattern is also observed in EAS Factor 3, in relation to the variable of having solid ethical training. Although this factor is not significant at the overall level of EAS and ATE, it demonstrates that a lower level of ethical training is associated with more positive attitudes toward euthanasia, particularly in the domain of “practical considerations.”

No statistically significant association was found between any of the scales or their dimensions and participation in assisted death in the clinical practice of professionals. This finding can be explained by the fact that patient care and their requests involve a deontological commitment rooted in normative ethics, which healthcare professionals must respect. Although conscientious objection can be exercised, the results suggest a tendency among professionals to refrain from judging patients’ decisions, respecting their autonomy [77].

Furthermore, the regression analysis does not identify the level of training as a predictor of attitudes toward euthanasia. The observed tendency for participants who perceive themselves as insufficiently trained to hold more positive attitudes toward euthanasia, although seemingly paradoxical, may be explained by several factors. First, bioethical dilemmas require a deep understanding of complex principles—such as the anthropological foundations of ethics, dignity, and quality of life—which training seeks to develop [78]. However, this increased awareness of the moral and legal complexities involved might lead trained professionals to adopt a more cautious or conservative stance.

Conversely, those without formal ethics or euthanasia training may hold a more simplified or idealized view, resulting in more favorable attitudes. Additionally, personal values and experiences often shape attitudes before professional training, and these beliefs can be resistant to change through education alone. Thus, ethics training might not drastically alter pre-existing attitudes but instead provide tools for critical reflection and nuanced understanding. This complexity highlights the need for further research to explore how training interacts with pre-existing values and how educational programs can better address these dynamics.

Moreover, professionals may consider themselves poorly trained in this regard, although their professional experience already provides them with some tools for deliberative processes. This finding may also be influenced by the fact that attitudes and values regarding euthanasia can be formed before professional training and remain relatively stable even after formal education on the subject [75, 76].

Understanding that formal ethics and euthanasia training may not directly change pre-existing attitudes but rather enhance critical thinking and ethical reflection points to the need for more comprehensive and ongoing education. Training programs could benefit from incorporating modules that address personal values, emotional aspects, and real-life case discussions to better engage healthcare professionals in complex ethical dilemmas.

The results of this study offer important insights that can inform both professional training and policy-making related to euthanasia. Understanding the sociodemographic and professional factors influencing attitudes—such as religiosity, specialization, and experience—allows educators to tailor training programs that address specific concerns and ethical challenges faced by healthcare providers. For instance, training could focus more on ethical deliberation and legal frameworks for professionals with less experience or without specialty training. Moreover, policymakers might consider these attitudinal differences to develop guidelines and support systems that facilitate respectful, patient-centered care while addressing the moral complexities encountered by practitioners. Ultimately, integrating these findings into training and policy can enhance the ethical competence and preparedness of healthcare professionals in contexts where euthanasia is legally regulated.

## Conclusion

Healthcare professionals’ attitudes toward euthanasia are influenced by multiple sociodemographic and experiential factors. This study identifies key variables shaping these attitudes, including religiosity, marital status, age, parenthood, having a close relative with a severe or incurable illness, recent bereavement, knowledge of euthanasia legislation, and ethical training. Among these, religiosity emerges as the most significant predictor of attitudes toward euthanasia.

For the ATE scale, religiosity and having a professional specialty emerged as significant negative predictors, with religious professionals and specialists showing less favorable attitudes toward euthanasia. Variables such as age, sex, marital status, parenthood, and years of professional experience were not significant predictors for ATE scores.

In contrast, for the EAS scale, religiosity was again the strongest negative predictor. Having a professional specialty also negatively influenced attitudes. However, unlike in the ATE, age and years of professional experience showed a small but significant positive association with more favorable attitudes toward euthanasia. Sex, marital status, and parenthood were not significantly associated with attitudes as measured by EAS.

These findings suggest that religiosity and specialization consistently relate to more conservative attitudes toward euthanasia, while age and professional experience may foster more positive attitudes when measured by EAS. Understanding these nuanced differences between scales can help tailor training and policy interventions to better address healthcare professionals’ views on euthanasia in the Spanish context. While both the EAS and ATE scales correlate in measuring euthanasia attitudes, they differ in their psychometric properties and in how they capture the factors that modulate these attitudes.

Assessing and understanding healthcare professionals’ attitudes in the Spanish context, especially after the enactment of euthanasia legislation, provides important insights. These findings can guide targeted interventions, educational programs, and policy improvements to ensure that euthanasia practices are applied ethically and effectively.

### Directions for future studies

Future research should explore in greater depth how specific components of professional training—such as bioethics education, legal knowledge, and practical experience—influence healthcare professionals’ attitudes and decision-making regarding euthanasia. Longitudinal studies could help determine whether attitudes change over time with increased exposure and education. Additionally, qualitative research could provide richer insights into the underlying reasons behind the paradoxical finding that less trained professionals show more favorable attitudes, exploring personal values, cultural influences, and ethical reasoning processes. Investigating the impact of different specialties and healthcare settings on attitudes would also be valuable to design more targeted educational interventions and policy measures.

### Strength and limitations

This study presents several strengths. It includes a relatively large and diverse sample of healthcare professionals from various disciplines working in the Balearic Health Service, which increases the relevance and applicability of the findings within that healthcare context. Additionally, the use of validated and culturally adapted instruments ensures the quality and consistency of the data collected. Importantly, this research addresses a highly pertinent and timely topic, as it explores healthcare professionals’ attitudes and knowledge following the recent implementation of the euthanasia law in Spain, providing valuable insights during this transitional period.

However, the study also has several limitations that should be acknowledged. First, as with all cross-sectional surveys relying on self-reported data, there is a potential for response bias, particularly regarding socially sensitive topics such as attitudes toward euthanasia and assisted suicide. Second, although the sample was large and included a wide range of healthcare professionals, the use of a non-probabilistic, voluntary response sample limits the generalizability of the results to all healthcare professionals in the region. Although the sample included a variety of healthcare professionals from different fields, participation was voluntary and may not fully represent the overall population of healthcare professionals within the Balearic Health Service. Second, the response rate, although acceptable for online surveys, was relatively low, and no information was available on the characteristics of non-respondents. Third, the cross-sectional design prevents establishing causal relationships.

## Data Availability

The data that support the fndings of this study are available from the author, Dr. María Dolores Onieva-Zafra (mariadolores.onieva@uclm.es), upon reasonable request.
